# Persistent Pulmonary Hypertension of Non Cardiac Cause in a Neonatal Intensive Care Unit

**DOI:** 10.1155/2012/818971

**Published:** 2012-05-09

**Authors:** Gustavo Rocha, Maria João Baptista, Hercília Guimarães

**Affiliations:** ^1^Division of Neonatology, Department of Pediatrics, Hospital de São João EPE, Faculty of Medicine of Porto University, 4202-451 Porto, Portugal; ^2^Division of Pediatric Cardiology, Department of Pediatrics, Hospital de São João EPE, Faculty of Medicine of Porto University, 4202-451 Porto, Portugal

## Abstract

Parenchymal lung diseases are the main cause of persistent pulmonary hypertension of the newborn (PPHN). We aimed to assess the non cardiac conditions associated to PPHN in the newborn and the survival rate over the last 15 years, at our center. A retrospective chart review of the neonates admitted for PPHN from 1996 to 2010 was performed. New therapies were introduced in 2003, and the survival rates between two periods (1996–2002 and 2003–2010) were compared. Out of 6750 newborns, 78 (1.1%) had the diagnosis of PPHN of non cardiac cause. The most prevalent causes were associated to pulmonary hypoplasia (30.7%), infection (24.3%), and aspiration syndromes (15.3%). Many other causes were identified in 33.3%. The overall survival rate was 68%. There was a significant difference on survival rates between the two periods (1996–2002 = 63.8% and 2003–2010 = 71.4%, *P* = 0.04). Our study showed a myriad of non cardiac aetiologies for PPHN of the newborn, most of them related to lung disease or lung hypoplasia. We observed an improvement in survival rate since 2003, which was associated to the use of new therapies.

## 1. Introduction

From the first clinical classification of pulmonary hypertension (PH), in Evian (France) in 1973, the knowledge about the disease significantly improved and recently, in 2008, that classification was updated at Dana Point (USA) [1]. This classification tries to include all possible causes of PH in children and adults; nevertheless, it is not a specific classification for PH presenting in the newborn.

PH presenting in the neonatal period may result from a myriad of causes [2]. Most commonly, it presents immediately after birth, a condition referred to as persistent pulmonary hypertension of the newborn (PPHN), when pulmonary vascular resistance fails to decrease at birth. This disease is recognized as arterial PH in the Dana Point classification of PH. Most cases of PPHN are associated with lung parenchymal diseases, such as meconium aspiration syndrome, and respiratory distress syndrome; however, some present without known lung disease as primary PPHN. Some infants who have PPHN have lethal causes of respiratory failure, such as alveolar-capillary dysplasia [3], genetic defects in surfactant synthesis [4], or severe lung hypoplasia secondary to oligohydramnios or congenital anomalies. Congenital heart diseases are also a possible cause of PH, but usually the prognosis and outcome are more related to the heart disease than to the pulmonary vascular involvement during the first weeks of life. In a new group of newborns, PH presents without known heart or lung disease, as primary PPHN.

Over the last decades, a timely referral to a tertiary centre, the use of new techniques of mechanical ventilation, extracorporeal membrane oxygenation, a better support therapy, the use of inhaled nitric oxide (iNO), and new pharmacological pulmonary vasodilators have ameliorated the prognosis of this clinical condition allowing a survival rate of about 90% in several referral centres [5].

The aims of this study were to review the non cardiac conditions associated to PPHN in the newborn and the survival rate of the affected patients over the last 15 years, at our centre.

## 2. Material and Methods

Neonates with the diagnosis of PPHN of non cardiac cause, admitted between 1996 and 2010, were identified from the database of our neonatal intensive care unit (NICU), a tertiary referral center for neonatal cardiac and pediatric surgery in the north of Portugal. Gestational data, demographic data, the cause of PPHN, treatment, days of NICU stay, neonatal outcome, and necropsy findings of the deceased neonates were retrieved from the clinical charts and retrospectively reviewed.

The diagnosis of PPHN was made on clinical grounds, chest X-ray, arterial blood gases analysis, and 2D-echocardiograhic findings. Pulmonary artery pressure estimation was based on the gradient between right ventricle and atrium, through tricuspid regurgitation, assuming the right atrium pressure as 15 mmHg (estimated pulmonary systolic artery pressure (PSAP) = right ventricle to right atrium gradient + 15 mmHg).

The diagnosis of PPHN was made on clinical grounds, chest X-ray, arterial blood gases analysis, and 2D-echocardiograhic findings. Pulmonary artery pressure estimation was based on the gradient between right ventricle and atrium, through tricuspid regurgitation, assuming the right atrium pressure as 10 mmHg (estimated pulmonary systolic artery pressure (PSAP) = right ventricle to right atrium gradient + 15 mmHg). Pulmonary hypertension was stratified as mild if estimated PSAP was less than 40 mmHg, moderate if between 40 and 60 mmHg, and severe if higher than 60 mmHg. Additionally, other parameters were evaluated to help in definition of the severity of PH: (i) shunt direction at ductus arteriosus or foramen ovale (left-to-right shunt was considered normal, bidireccional shunt was considered sign of mild to moderate PH and right to left shunt was considered sign of severe PH); (ii) orientation of ventricular septum (the normal orientation was considered left to right, septum rectification was indicative of mild-to-moderate PH, and when the septum budge from right-to-left a severe PH was likely), and (iii) systolic function of the left ventricle, through the left ventricular ejection fraction (in cases of moderate PH it was expected a hipercontractil left ventricle whilst in severe PH usually we found a decrease on left ventricle ejection fraction). All the parameters were evaluated routinely. Echocardiographic evaluation was also used to exclude or confirm any congenital heart disease.

Inhaled nitric oxide (iNO) (usually starting with 20 ppm) has been routinely used since 2003 after echocardiographic definition of severe PH and an oxygenation index (mean airway pressure × fraction of inspired oxygen × 100/partial arterial pressure of oxygen) over 20. Sildenafil has been used in infants with persistent pulmonary hypertension refractory to iNO. Since iNO and sildenafil have been used since 2003, a comparison of the survival rates between two epochs was made (1996–2002 and 2003–2010).

Since 2003, we have also routinely used a total daily water intake of 80 mL/kg (until start enteral feeds) along with a perfusion of dopamine 5 mcg/kg/min, in order to keep a systemic blood pressure over 40 mmHg, and a hematocrit of about 45% (haemoglobin ≥15 g/dL). A perfusion of dobutamine (5 mcg/kg/min) is started if signs of myocardial dysfunction are present at echocardiographic evaluation. Higher doses of dopamine and dobutamine or epinephrine perfusion are used according to clinical criteria. Minimal stimulation as well as sedation and analgesia is usually performed with a perfusion of morphine (or fentanyl in the case of hypotension) and midazolam. Paralyzing agents as vecuronium are usually avoided; it is only used in selected cases as a rescue ventilation adjunt therapy. When mechanical ventilation is need, conventional ventilation is preferred to high-frequency oscillation ventilation, which is mainly used as rescue ventilation. The goals of mechanical ventilation are to maintain a PaO_2_ of 60–90 mmHg and a PaCO_2_ >30 mmHg (usually 35–50 mmHg), in order to avoid oxidative stress and hypocapnia.

ECMO treatment was not achievable in our country until 2010. Our centre, recently, started ECMO support to neonates and children.

Categorical variables were compared through Chi-square or the exact Fisher's test. The Mann-Whitney test was used to compare two independent samples.

This study has been approved by the ethics committee board of our institution.

## 3. Results

In the last 15 years, 6750 newborns were admitted to our unit. Seventy-eight (1.1%) had the diagnosis of PPHN of non cardiac cause. The demographics of the studied population are reported in Table 1, and the causes of PPHN are reported in Table 2. Twenty-five (32.0%) were deceased (13 males; 12 females). The median of death day was 7 (1–114). There were 34 outborns that were referred to our centre. Mortality rate in the outborn group was 32.3% (11/34), not different from the inborn group that was 31.8% (14/44) (*P* = 0.967). Pulmonary hypertension was classified as mild in 14 (17.9%) patients, moderate in 24 (30.7%), and severe in 40 (51.2%). Treatment aspects are reported in Table 3. The normalization of pulmonary hypertension occurred by day eight of life (2–160) in the survivors. The median of days of stay in the NICU was 12 days (1–167). The overall survival rate was 68%. There was a significant difference on survival rates between two periods (1996–2002 = 63.8% and 2003–2010 = 71.4%) (*P* = 0.04); see Table 4. Along with this increase in survival, days of NICU stay and of normalization of PH in the survivors accordingly increased.

## 4. Discussion

Persistence of pulmonary hypertension leading to respiratory failure in the neonate has been recognized for 40 years since its original description by Gersony and colleagues in 1969 [6]. During the development of the pulmonary vasculature in the fetus, many structural and functional changes occur to prepare the lung for the transition to air breathing. The development of the pulmonary circulation is genetically controlled by an array of mitogenic factors in a temporospatial order. With advancing gestation, pulmonary vessels acquire increased vasoreactivity. The fetal pulmonary vasculature is exposed to a low oxygen tension environment that promotes high intrinsic myogenic tone and high vasocontractility. At birth, a dramatic reduction in pulmonary arterial pressure and resistance occurs with an increase in oxygen tension and blood flow. The striking hemodynamic differences in the pulmonary circulation of the fetus and newborn are regulated by various factors and vasoactive agents. Among them, nitric oxide, endothelin-1, and prostaglandin I(2) are mainly derived from endothelial cells and exert their effects via cGMP, cAMP, and Rho kinase signalling pathways. Alterations in these signalling pathways may lead to vascular remodelling, high vasocontractility, and PPHN [7, 8].

In this study we were able to document PPHN in 16 preterm neonates, including one with 30 weeks of gestational age with a congenital sepsis and pneumonia. It is already known that the mechanisms that could lead to PH are already present in the human fetus by 31 weeks of gestation [5, 9, 10]. In our patients we observed a high number of C-sections that are related to prenatal diagnosis of pulmonary hypoplasia, as congenital diaphragmatic hernias, Potter syndrome, or meconium-stained amniotic fluid.

The most common cause of PPHN in this study was pulmonary hypoplasia. Congenital diaphragmatic hernia was the most prevalent cause of pulmonary hypoplasia. Congenital diaphragmatic hernia and oligohydramnios secondary to renal anomalies or premature rupture of membranes leads to pulmonary hypoplasia. Pulmonary hypertension often occurs as a complication because of the decreased number of blood vessels and increased reactivity of the vessels in the hypoplastic lungs. PPHN is usually more chronic and less responsive to vasodilator therapy in these infants and their outcome is related to the degree of lung hypoplasia, associated anomalies, as well as lengt of pulmonary hypertension [11]. The outcome for neonates who have congenital diaphragmatic hernia has improved since gentle ventilation and permissive hypercapnia have been incorporated into the management, with many centers reporting 75% survival in recent years [11, 12]. The survival of patients with congenital diaphragmatic hernia has improved in our center since 2003, and is nowadays over 61% [13].

The second cause of PPHN in this study was congenital pneumonia and sepsis. PPHN can be a complication of pneumonia or sepsis secondary to common neonatal pathogens [14]. Bacterial endotoxin causes pulmonary hypertension from several mechanisms, including the release of thromboxane, endothelin, and several cytokines [15, 16]. Sepsis also leads to systemic hypotension from activation of inducible nitric oxide synthase with excess nitric oxide release in the systemic vascular beds, impaired myocardial function, and multiorgan failure. Addressing PH should be a component of the overall management of septic shock and prevention of multiorgan failure in the affected neonates.

Another significant group of causes of PPHN were the aspiration syndromes, mainly meconium aspiration syndrome, representing 12.8% of PPHN in this series. Although meconium staining of amniotic fluid occurs in 10% to 15% of pregnancies, meconium aspiration syndrome occurs infrequently, in up to 5% of neonates born through meconium stained fluid. The incidence of meconium aspiration syndrome has declined in recent years [17] with decreasing postterm pregnancies. This observation suggests that meconium aspiration syndrome is often a result of in utero stress with aspiration of meconium by a compromised fetus. Meconium can cause respiratory failure from several mechanisms. Meconium can cause mechanical obstruction to the airways, particularly during exhalation, resulting in air trapping, hyperinflation, and increased risk for pneumothorax. Meconium components also inactivate surfactant, [18] trigger an inflammatory response with release of cytokines, and increase the production of the vasoconstritors endothelin and thromboxane [19]. Recent advances in the management of PPHN have resulted in an excellent outcome for neonates who have meconium aspiration syndrome [20].

In this study PPHN occurred as a complication of hyaline membrane disease and transient tachypnea of the preterm newborn, often delivered by C-section, at 34–37 week's of gestation. The increasing reactivity of pulmonary arteries at this gestation period predisposes these neonates to PH when gas exchange is impaired because of surfactant deficiency [21].

The association of PPHN with maternal intake of nonsteroid anti-inflammatory drugs as indomethacin has been recognized in case reports since 1970 [22, 23]. A strong causal association is also suggested by the consistent reproduction of hemodynamic and structural features of PPHN by fetal ductal constriction [24, 25].

Maldevelopment of pulmonary arteries (pulmonary “arteriopathy” and idiopathic pulmonary arteriolar calcification) and thrombosis of pulmonary arteries (probably associated to coagulation disorders that were not assessed) were necropsy findings in three patients without any evident cause for the PH on clinical grounds. Maladaptation of the pulmonary vascular bed in asphyxia, maternal diabetes, and fetal tachyarrhythmia were also identified in this study, as well as in two patients with malformation of vein of Galeno and PPHN associated to high cardiac output failure from large arteriovenous malformations. These causes of PPHN have already been described [26]. The cases of PPHN of unknown aetiology in this series were transient forms with a good outcome, suggesting transient maladaptation to extrauterine life.

Neonates who have PPHN require supportive care tailored to the degree of hypoxemia and physiologic instability. Oxygen is a potent vasodilator and was used in all patients, once hypoxemia is usually present. Mechanical ventilation facilitates alveolar recruitment and lung expansion, potentially improving the ventilation/perfusion (V/Q) match. In this study, mechanical ventilation was used in all, except in four cases of transient tachypnea of the newborn and three cases of PPHN of unknown aetiology with mild pulmonary hypertension. Surfactant has been shown to decrease the need for ECMO in full-term neonates with severe respiratory failure [27]. The beneficial effect of surfactant in this study was seen particularly in babies who had meconium aspiration syndrome and sepsis. Sedatives, although widely used to minimize fluctuations in oxygenation and facilitate ventilation, have not been tested in randomized trials [5]. We have used sedatives in all ventilated patients to ameliorate oxygenation and to decrease discomfort. We do not use for routine skeletal muscle relaxants. Inotropic and vasopressor support with dopamine, dobutamine, and epinephrine is used to optimize cardiac function, stabilize systemic blood pressure, and decrease right-to-left shunt. From 2003 we have used iNO and sildenafil, in selected cases of severe PPHN, mainly in congenital diaphragmatic hernia. Both the survival rate of congenital diaphragmatic hernia and all cases of PPHN showed a significant increase since 2003. ECMO has significantly improved the survival of neonates with severe but reversible lung disease [28, 29], but we do not have experience on that. We tried ECMO in a poor prognosis for of bilateral congenital diaphragmatic hernia with severe pulmonary hypoplasia and pulmonary hypertension, but the patient did not survive.

The overall survival rate described in the literature, including all causes of PPHN, is over 70–75% [30]. Our results are now according to these figures. There is, however, a marked difference depending on the underlying disease. There are also significant differences in long-term outcome according to the cause of PPHN.

This study is limited by the fact of being a single center retrospective analysis, including a small sample of some rare pulmonary disorders causing PH. Prospective studies with the objective of evaluating the different therapies in the various groups of underlying diseases, including a significant number of patients, will give much more information regarding therapeutic efficacy and survival.

Future research must address the different causes of PPHN and therapies separately, in large multicenter studies.

In conclusion, our study shows a myriad of non cardiac aetiologies for PPHN, most of them related to lung disease or lung hypoplasia. We observed an improvement in survival rate since 2003, and we believe that this is related to the use of new therapies. We hope that ECMO will offer additional advantages at our NICU for selected infants in the near future.

## Figures and Tables

**Table 1:** Demographics (*n* = 78).

Gestational age (weeks), median (min–max)	39 (30–41)
Preterm (<37 weeks gestation)	16 (20.5%)
Birthweight (grams), median (min–max)	3080 (1450–4170)
Intrauterine growth restriction	4 (5%)
Gender	
male	53 (67.9%)
female	25 (32.1%)
C-section	51 (65.3%)
Outborn	34 (43.5%)

**Table 2:** Causes of PPHN (*n* = 78).

Aspiration of bloody amniotic fluid, *n* (%)	1 (1.2)
Aspiration of blood from upper airways	1 (1.2)
(traumatic intubation), *n* (%)	
Meconium aspiration syndrome, *n* (%)	10 (12.8) (2^†^)
Congenital pneumonia and sepsis, *n* (%)	19 (24.3) (4^†^)
Severe hyaline membrane disease, *n* (%)	3 (3.8) (1^†^)
Transient tachypnea of the newborn, *n* (%)	4 (5.1)
Intrauterine ductus arteriosus closure	2 (2.5)
(indomethacin), *n* (%)	
Congenital diaphragmatic hernia, *n* (%)	17 (21.7) (10^†^)
Potter syndrome, *n* (%)	1 (1.2) (1^†^)
Nephrourological malformation with	1 (1.2)
oligoamnios, *n* (%)	
Idiopathic hypoplastic lung, *n* (%)	2 (2.5) (1^†^)
Idiopathic pulmonary arteriolar	1 (1.2) (1^†^)
calcification, *n* (%)	
Pulmonary “arteriopathy”, *n* (%)	1 (1.2) (1^†^)
Arterial pulmonary thrombosis, *n* (%)	1 (1.2) (1^†^)
Fetal tachyarrhythmia, *n* (%)	1 (1.2)
Maternal diabetes, *n* (%)	1 (1.2) (1^†^)
Malformation of vein of Galeno, *n* (%)	2 (2.5) (2^†^)
Perinatal asphyxia, *n* (%)	4 (5.1)
Unknown aetiology, *n* (%)	6 (7.6)

^†^: deceased.

**Table 3:** Treatment (*n* = 78).

Inhaled nitric oxide, *n* (%)	19 (24.3%)
Surfactant, *n* (%)	24 (30.7%)
Dopamine, *n* (%)	57 (73%)
Dobutamine, *n* (%)	35 (44.8%)
Epinephrine, *n* (%)	3 (3.8%)
Sildenafil, *n* (%)	12 (15.3%)
Diuretics, *n* (%)	33 (42.3%)
Sedation, *n* (%)	71 (91%)
Oxygen, *n* (%)	78 (100%)
Days of oxygen, median (min–max)	6 (1–114)
Mechanical ventilation, *n* (%)	71 (91%)
Days of mechanical ventilation, median (min–max)	7 (1–114)
Extracorporeal membrane	1 (1.2%)
oxygenation (ECMO), *n* (%)	
Days of ECMO	17

**Table 4:** Survival rates between two periods.

	1996–2002	2003–2010	*P*
	*n* = 36	*n* = 42	
Gestational age, weeks, median (min–max)	41 (30–41)	39 (32–41)	**0.032** ^§^
Preterm (<37 weeks of gestation), *n* (%)	8 (22)	8 (19)	0.081*
Birthweigt, g, median (min–max)	3100 (1450–4170)	3040 (1800–4070)	0.354^§^
Gender			
Male, *n* (%)	23 (64)	30 (71)	
Female, *n* (%)	13 (36)	12 (29)	0.456*
C-section, *n* (%)	23 (64)	28 (67)	0.657*
Outborn, *n* (%)	18 (50)	16 (38)	0.071*
NICU stay	10 (1–67)	16 (1–167)	**0.034** ^§^
Time to normalization of PH	5 (2–25)	9 (2–160)	**0.0391** ^§^
Survival, *n* (%)	23 (63.8)%	30 (71.4)	0.040**

^§^: Mann-Whitney test; *: Chi-Squared test; **: Fisher Exact test; NICU: neonatal intensive care unit; PH; pulmonary hypertension.
